# Volcanic SO_2_ total mass dataset on Mt. Etna (Italy) from 2018 to 2025 using sentinel-5P TROPOMI

**DOI:** 10.1016/j.dib.2026.112876

**Published:** 2026-05-20

**Authors:** Maddalena Dozzo, Alessandro Aiuppa, Giuseppe Bilotta, Annalisa Cappello, Gaetana Ganci

**Affiliations:** a Istituto Nazionale di Geofisica e Vulcanologia, OE 95125 Catania, CT, Italy; b Dipartimento di Scienze della Terra e del Mare (DiSTeM), Università degli Studi di Palermo 90123 Palermo, PA, Italy

**Keywords:** Google earth engine, Clustering analysis, Sentinel-5P, Sulfur dioxide, Volcanoes

## Abstract

Sulfur dioxide (SO₂) is released by the magma degassing in the shallow crust, constituting a key indicator of magma ascent rates in the feeding conduit, as well as providing information on the style and the intensity of eruptive activity. Continuous monitoring of this gas is important to understand volcanic processes and to contribute to hazard assessment. The dataset presented here provides a comprehensive time series of SO₂ total mass from Mount Etna (Sicily, Italy), covering the period from 2018 to 2025. The data have been obtained from the TROPOspheric Monitoring Instrument (TROPOMI) onboard the Sentinel-5 Precursor satellite, which has been operational since 2018, delivering atmospheric column measurements of sulfur dioxide and other gases at unprecedented spatial resolution and daily revisit time. Volcanic SO₂ plumes were automatically identified through a two-step procedure: firstly, the Simple Non-Iterative Clustering (SNIC) segmentation method was applied, which is an object-based image analysis technique and secondly, K-means unsupervised clustering was used on the segmented imagery to further improve cloud detection. The algorithm has been implemented in the open-source Google Earth Engine platform, enabling efficient processing of the TROPOMI imagery collection, to which quality control filters are already applied. This methodological framework supports the generation of SO₂ total mass time series with reduced delay and improved calculation time, thus providing a valuable tool for rapid and reliable monitoring of volcanic emissions and for enhancing volcanic hazard assessment capabilities.

Specifications Table

**Table:** 

Subject	Earth & Environmental Sciences
Specific subject area	Retrieval and quantification of sulfur dioxide from volcanic sources using satellite data and clustering techniques.
Type of data	Table (.csv format)
Data collection	Both Level 2 and Level 3 Sentinel-5P TROPOMI Offline products were used. The first one, used only for validation, was collected from the Copernicus Browser, whereas the Sentinel-5P TROPOMI Level 3 dataset, used in the computation of the SO_2_ masses, was provided by the open access Google Earth Engine platform. A geographical filter was applied to the area around Etna volcano, and a temporal filter was defined to select the period from 2018 to 2025. To select only pixels containing SO_2_ on Level 2 data, the relative variable (“sulfurdioxide_detection_ flag”) was set to a value higher than 0.
Data source location	The study area is around Mt. Etna volcano (Sicily, Italy) and is defined by a bounding box with corner coordinates (latitude, longitude) in WGS84: southwest (32.76° N, 7.78° E) and northeast (41.50° N, 22.98° E).
Data accessibility	Repository name: Figshare Data identification number: https://doi.org/10.6084/m9.figshare.31313977 Direct URL to data: https://doi.org/10.6084/m9.figshare.31313977
Related research article	Dozzo, M., Aiuppa, A., Bilotta, G., Cappello, A., & Ganci, G. (2025). A New Algorithm for the Global-Scale Quantification of Volcanic SO2 Exploiting the Sentinel-5P TROPOMI and Google Earth Engine. Remote Sensing, 17(3), 534. https://doi.org/10.3390/rs17030534.

## 1 Value of the Data

• This dataset contains daily values of the total SO₂ mass from Mt. Etna (expressed in kilotons), derived from Sentinel‑5P TROPOMI Level 3 satellite imagery and processed through a standardized object‑based detection workflow [1]. It delivers a consistent, high‑resolution time series from 2018 to 2025, while removing the need for manual data retrieval and complex pre‑processing steps. The latter include the application of quality filters, background corrections, and the conversion from Slant Column Density to Vertical Column Density (VCD) using the Air Mass Factor, whereas Google Earth Engine directly provides VCD values.
• The presented time series of SO₂ total mass constitutes a valuable dataset, as no similar data collections are currently available in the literature. Such information is crucial for immediate assessments of volcanic hazard estimates.
• The developed algorithm is straightforward and reproducible and, in principle, could be applicable to different gas species or emission sources, including those of anthropogenic origin. This step can be easily done by defining the area of interest and the type of gas, directly on Google Earth Engine.
• The dataset can be reused by researchers to track volcanic gas emissions, compare eruptive activity across different regions, and include the data in atmospheric transport or dispersion models.
• The data supports cross‑comparison with ground‑based measurements and other satellite products, enabling validation studies and multi‑sensor data integration for volcanic gas monitoring at global scale.

## 2 Background

The dataset was compiled to enable rapid, global-scale quantification of volcanic SO₂ emissions using Sentinel-5P TROPOMI Level 3 products within the Google Earth Engine (GEE) platform. The motivation comes from the need to process large volumes of satellite data efficiently, without manual downloading or complex pre-processing, and to address the lack of an SO₂ identification mask in Level 3 products. Moreover, the calculation of SO₂ total mass by applying a simple fixed threshold directly to the TROPOMI product does not allow obtaining optimal results [2].

The methodological framework combines object-based image analysis through the Simple Non-Iterative Clustering (SNIC) segmentation algorithm and the k-means unsupervised classifier to automatically retrieve volcanic SO₂ plumes, applying a consistent density threshold to isolate SO₂-contaminated pixels. The dataset includes a time series of SO₂ total mass for Mount Etna from 2018 to 2025, computed from daily TROPOMI imagery. This data article supports the related research publication by providing the processed SO₂ mass values derived from the described algorithm.

## 3 Data Description

The file associated with this data-in-brief article is the following:

SO2 Total Mass on Etna.csv: The data file contains 2584 dates (from the end of 2018 to the end of 2025), in the format dd/mm/yyyy. Each date is associated with the corresponding SO₂ total mass value, which is expressed in kilotons (kton). These values represent those retrieved on Level 3 Offline TROPOMI product on the Google Earth Engine Platform, after the application of the two clustering techniques (SNIC and k-means) and the density threshold, in the area of interest defined around Mt. Etna.

The collection used for the creation of the dataset is the gridded Level 3 Sentinel-5P TROPOMI. This product is already set on the GEE Platform e ready for use. It is obtained from the original Level 2 SO₂ data from the European Space Agency and re-gridded and merged using the HARP (Harmonized Access to Remote Data for Processing) software, ensuring a uniform global resolution. During re-gridding, HARP also applies quality assurance (QA) filtering to remove low-quality pixels affected by clouds, snow, or ice, with product-specific criteria to retain only high-confidence observations. The final dataset has a spatial resolution of approximately 0.1° × 0.1°.

QA filtering criteria applied by ESA within the HARP processing procedure, reporting the name of each variable as indicated in Google Earth Engine, include:

• snow_ice < 0.5 (Removes pixels significantly covered by snow or ice. High surface albedo in UV strongly distorts the radiative transfer and leads to overestimation of SO₂ columns).
• sulfurdioxide_total_air_mass_factor_polluted > 0.1 (Keeps only observations with a sufficiently large Air Mass Factor (AMF). Indeed, very small AMF values indicate low measurement sensitivity to SO₂ (for example due to an unfavorable viewing geometry, low plume altitude, or cloud/aerosol shielding), making the retrieved vertical column density unreliable).
• sulfurdioxide_total_vertical_column > −0.001 mol/m² (Filters out strongly negative SO₂ values, which mainly represent noise when the SO₂ signal is below the instrument detection limit).
• qa_value > 0.5 (General quality assurance flag (0 = failed retrieval, 1 = optimal). This setting, which is the recommended threshold for scientific use, removes measurements affected by fitting errors, radiometric issues, clouds, or poor observing conditions.
• cloud_radiance_fraction < 0.3 (Selects mostly clear-sky pixels. Indeed, clouds can alter the photon path length and could either shield the plume or artificially amplify the signal, causing large errors in SO₂ retrieval).
• solar_zenith_angle < 60° (Excludes low-sun conditions. Large solar zenith angles increase atmospheric scattering and reduce signal-to-noise ratio, lowering the degree of SO₂ retrieval accuracy).

The GEE platform provides two different Level 3 TROPOMI products, namely Sentinel-5P Offline Sulfur Dioxide and Sentinel-5P Near-Real-Time Sulfur Dioxide. This second product is designed for near-real-time applications and remains available for 15 days after acquisition, after which it is merged into and replaced by the Offline product.

## 4 Experimental Design, Materials and Methods

**Fig. 1:**
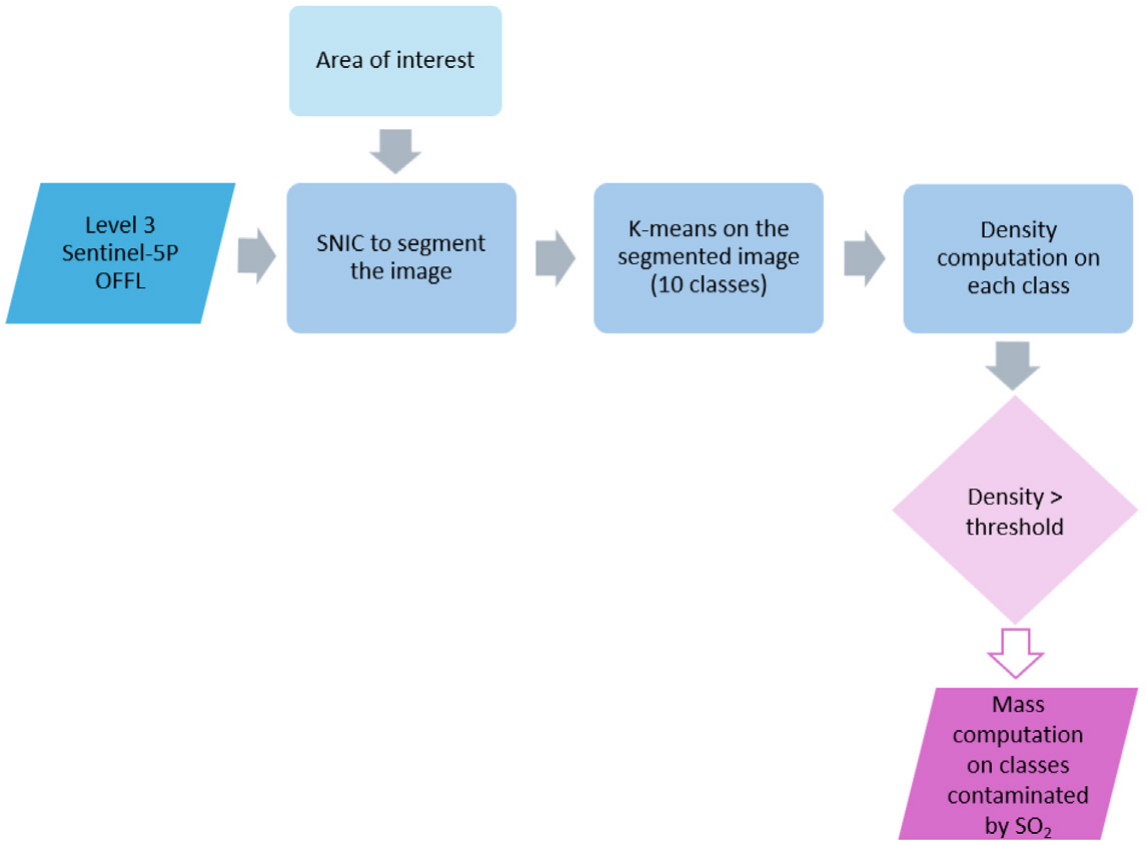
Flowchart with the main steps of the methodology: the Level 3 Sentinel-5P Offline Product and the geometry outlined around Etna represent the input data; light blue rectangles report the main processing steps (where SNIC stands for Simple Non-Iterative Clustering), while the purple parallelogram represents the output to highlight the pixels contaminated by SO₂ within those classes with a density higher than a specific threshold. The SO₂ total mass was computed only on these pixels.

In Fig. 1 is represented the workflow followed to obtain the final SO₂ total masses dataset.

To accurately isolate pixels containing SO₂ and prevent the inclusion of noisy artifacts with artificially high SO₂ values outside the actual plume, two clustering techniques were applied to all pixels within a geometry centered on the volcano, large enough to cover most of the plume. The Sentinel‑5P Offline Sulfur Dioxide images in GEE were first segmented using the SNIC algorithm, followed by a k‑means unsupervised classification. This sequential, object‑based image analysis approach enables a more precise characterization of SO₂ while reducing the impact of noise caused, for instance, by satellite sensor artifacts or variations in lighting and atmospheric conditions [3].

Image segmentation divides an image into multiple coherent regions. Advanced methods such as Multi‑Resolution Segmentation (MRS) [4], Simple Linear Iterative Clustering (SLIC) [5], and its non‑iterative variant SNIC [6] have shown improved accuracy and reduced processing time compared to traditional pixel‑based classifications, especially for high‑resolution remote sensing data. SNIC produces superpixels with strong boundary adherence and low adjacency, offering advantages in memory efficiency and speed [7]. The SNIC algorithm starts by placing K centroids on a regular grid, each linked to spatial position, label, and distance from its centroid [8]. Pixels are processed in order of increasing distance using a priority queue. Each unclassified pixel is assigned a label, and centroid values are updated until all pixels have been labelled [9]. SNIC requires setting key parameters: compactness (which affects the shape of the clusters), connectivity (4 or 8, which controls the cohesion), and neighborhood size (that represents the number of pixels composing each superpixel). For this application, values were set to compactness = 20, connectivity = 4, and neighborhoodSize = 20, selected through a trial‑and‑error analysis and visual inspection to balance detail preservation, segmentation clarity, and computational efficiency. The parameter values were checked by comparing them with the k-means output to ensure the best results.

The k‑means unsupervised clustering algorithm was applied to the SNIC‑segmented images to assign all pixels within each cluster to the same class, thereby refining plume identification and improving classification clarity. Running k‑means on superpixels reduces computational load by working on fewer objects than in the original image. K‑means operates iteratively: k initial centers are randomly set, each pixel is assigned to its nearest center (based on Euclidean distance [10]), and cluster means are recalculated until convergence to a local minimum [11]. A k value equal to 10 was selected to provide a detailed classification of SO₂ concentration levels.

The volcanic plume was identified by selecting the classes with the highest SO₂ density, calculated as the ratio between the total SO₂ mass (sum of SO₂ values for all pixels in a class) and the number of pixels within that class. This approach enabled the computation of total SO₂ mass considering only pixels that form the plume. A density threshold of 0.00028 mol/m² was selected, as it provided the best separation between SO₂‑contaminated and background pixels across multiple eruptive events. This threshold value allowed the volcanic cloud to be defined by a few continuous classes, and the total SO₂ mass was then computed on each plume and expressed in kton.From the end of 2018 to the end of 2025, several eruptive events occurred at Mount Etna.

The period 2018–2019 was affected by several episodes. In particular, a major event occurred on 24–27 December 2018, known as the “Christmas eruption.” During that period, after approximately 15 months of quiescence following the February–April 2017 eruption, mild Strombolian activity and small lava flows resumed at the Bocca Nuova (BN) and North-East Crater (NEC) in July 2018, culminating in an eruptive episode in late August at the New South-East Crater (NSEC) [12].

During 2021 more than 60 paroxysmal events occurred at Etna at the South-East Crater (SEC) [13], producing high eruptive columns, often more times within a day and tons of SO₂. The eruptive sequence concluded in February 2022, with two particularly violent explosive events occurring on 10 and 21 February.

Effusive activity followed between 11 May and 13 June 2022, initially from high vents on SEC’s north flank (11–29 May), and later from two fissures in the Valle del Bove. Another long-lasting flank effusion took place from fissures in the Valle del Leone between 27 November 2022 and 6 February 2023 [14].

In 2023, four SEC strong events occurred: 21 May, 13–14 August, 12 November, and 1 December, producing lava fountains and flows. The 21 May 2023 event also generated a pyroclastic density current [15].

After more than four years of quiescence, the Voragine (VOR) crater of Mount Etna reactivated on 14 June 2024 with weak Strombolian explosions and minor lava effusion into the BN crater. Overall, seven paroxysmal events (six between July and August and one in November) significantly reshaped Etna’s summit area, producing western BN overflows and complex, multi-directional lava flow fields [16].

In February 2025, Etna entered a new eruptive phase characterized by both effusive and explosive activity. Explosive activity began on 6 February with intermittent Strombolian eruptions from a vent in the western sector of the South-East Crater (SEC). Two days later, on 8 February, an effusive eruption started from a fissure at the base of the BN crater at about 3050 m a.s.l. The lava flow moved rapidly southwest, descending to ∼ 2150 m a.s.l. by 11 February and ∼ 1960 m a.s.l. by 13 February [17].

**Fig. 2:**
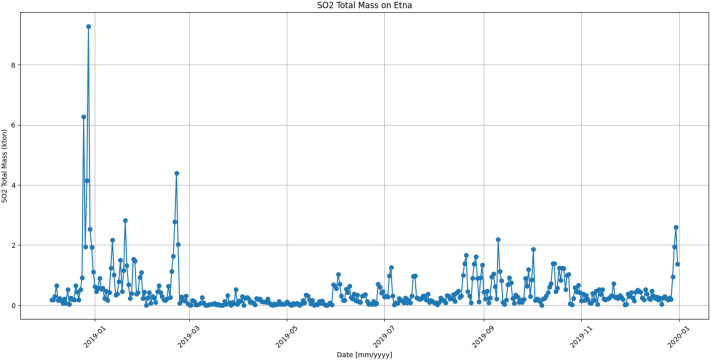
SO_2_ total mass time series computed in the area defined around Etna in the period from the end of 2018 to the end of 2019.

Fig. 2 reports the time series related to the SO_2_ total mass retrieved on Etna for the period from the end of 2018 (when the Christmas eruption occurred) to the end of 2019, taken as an example.

The major eruptive episode occurred on 24-27 December 2018 is reflected in the high SO_2_ values detected. Other significant peaks were recorded in the first months of 2019 and from late May onwards, evidence of episodes of intense degassing and eruptions, sometimes associated with paroxysmal events. After the resumption of eruptive activity in May, the activity remained almost constant, as described in the INGV-OE bulletins (www.ct.ingv.it; accessed on 11 February 2026).

Since the Level 3 TROPOMI dataset does not include a mask for SO₂-positive pixels (available only in Level 2), the Level 2 product was used for comparison. Level 2 data, obtained in netCDF-4 format from the ESA Copernicus Sentinel-5P Pre-Operation Data Hub (https://sentinel.esa.int/documents/247904/3541451/Sentinel-5P-Sulphur-Dioxide-Readme.pdf; accessed on 11 February 2026), are derived from raw Level 0 measurements that are calibrated, georeferenced, and processed to Level 1 radiance and irradiance. SO₂ concentrations are retrieved from the ultraviolet (UV) spectrum through differential optical absorption spectroscopy (DOAS) algorithms, using fitting windows of 312–326, 325–335, or 360–390 nm [18]. For volcanic pixels, the “sulfurdioxide_detection_flag” > 0 is applied [19], with flag values indicating: 0 = no detection, 1 = SO₂ detection, 2 = clear volcanic detection, 3 = near an anthropogenic source, and 4 = high solar zenith angle (possible false positive). By summing the pixels of the volcanic SO_2_ detection mask, the total mass of volcanic SO_2_ plumes in the image can be estimated [20]. The resulting SO₂ masses from Level 3 were validated against the Level 2 product, by comparing both the spatial distribution of the plume and the corresponding SO_2_ total amount, finding a strong agreement.

## Limitations

The dataset is derived exclusively using Sentinel‑5P TROPOMI Level 3 products at a fixed plume height of 15 km, as provided on the Google Earth Engine platform. In standard SO₂ retrieval operation, interpolation between different plume altitudes (typically 1 km, 7 km, 15 km) is performed to better approximate the real vertical position of the volcanic cloud. This step is important since the retrieved SO₂ column amount strongly depends on the plume height. Indeed, an incorrect assumption of the altitude may affect the air mass factor, leading to under‑ or overestimation of the total mass. The absence of the 7 km altitude layer in GEE, could limit accurate quantification for lower volcanic plumes, as interpolation between heights is not possible in the current dataset. Despite this limitation, the dataset provides a consistent and reproducible record of SO₂ total mass at daily resolution, suitable for long‑term volcanic monitoring. In the near future, this problem should be overcome as Google Earth Engine integrates the Sentinel‑5P Level 3 product at 7 km plume height, an update that will enable the interpolation between 1, 7, and 15 km layers. In this way, the new dataset can be included in the algorithm developed in this work, allowing to improve the accuracy for the low volcanic plumes.

## Ethics Statement

The authors confirm that they have read and follow the ethical requirements for publication in Data in Brief and that the current work does not involve human subjects, animal experiments, or any data collected from social media platforms.

## CRediT Author Statement

**Maddalena Dozzo:** Data Curation**,** Formal Analysis, Investigation, Methodology**,** Software, Visualization, Writing - Original Draft, Writing - Review & Editing. **Alessandro Aiuppa:** Resources**,** Supervision, Validation**,** Writing - Review & Editing. **Giuseppe Bilotta:** Formal Analysis, Investigation, Writing - Review & Editing. **Annalisa Cappello:** Investigation, Validation**,** Writing - Review & Editing. **Gaetana Ganci:** Conceptualization, Methodology, Resources, Supervision, Writing - Original Draft, Writing - Review & Editing.

## Declaration of Competing Interest

The authors declare that they have no known competing financial interests or personal relationships that could have appeared to influence the work reported in this paper.

## Data Availability

FigshareVolcanic SO2 Total Mass computed on Mount Etna (Sicily, Italy) exploiting Sentinel-5P TROPOMI satellite (Original data)
